# Inverse Agonism of SQ 29,548 and Ramatroban on Thromboxane A2 Receptor

**DOI:** 10.1371/journal.pone.0085937

**Published:** 2014-01-23

**Authors:** Raja Chakraborty, Rajinder P. Bhullar, Shyamala Dakshinamurti, John Hwa, Prashen Chelikani

**Affiliations:** 1 Department of Oral Biology, University of Manitoba, Winnipeg, Manitoba, Canada; 2 Departments of Pediatrics, Physiology, University of Manitoba, Winnipeg, Manitoba, Canada; 3 Biology of Breathing Group- Manitoba Institute of Child Health, Winnipeg, Manitoba, Canada; 4 Department of Internal Medicine (Cardiology), Cardiovascular Research Center, Yale University School of Medicine, New Haven, Connecticut, United States of America; University of North Dakota, United States of America

## Abstract

G protein-coupled receptors (GPCRs) show some level of basal activity even in the absence of an agonist, a phenomenon referred to as constitutive activity. Such constitutive activity in GPCRs is known to have important pathophysiological roles in human disease. The thromboxane A2 receptor (TP) is a GPCR that promotes thrombosis in response to binding of the prostanoid, thromboxane A2. TP dysfunction is widely implicated in pathophysiological conditions such as bleeding disorders, hypertension and cardiovascular disease. Recently, we reported the characterization of a few constitutively active mutants (CAMs) in TP, including a genetic variant A160T. Using these CAMs as reporters, we now test the inverse agonist properties of known antagonists of TP, SQ 29,548, Ramatroban, L-670596 and Diclofenac, in HEK293T cells. Interestingly, SQ 29,548 reduced the basal activity of both, WT-TP and the CAMs while Ramatroban was able to reduce the basal activity of only the CAMs. Diclofenac and L-670596 showed no statistically significant reduction in basal activity of WT-TP or CAMs. To investigate the role of these compounds on human platelet function, we tested their effects on human megakaryocyte based system for platelet activation. Both SQ 29,548 and Ramatroban reduced the platelet hyperactivity of the A160T genetic variant. Taken together, our results suggest that SQ 29,548 and Ramatroban are inverse agonists for TP, whereas, L-670596 and Diclofenac are neutral antagonists. Our findings have important therapeutic applications in the treatment of TP mediated pathophysiological conditions.

## Introduction

Thromboxane A2 (TXA2) is a major product of arachidonic acid metabolism and is known to be the key mediator of platelet aggregation and smooth muscle contraction [1], [2], [3]. The action of TXA2 is mediated by its cognate G protein-coupled receptor (GPCR) thromboxane A2 receptor (TP), which exists in two isoforms, TPα and TPβ, differing only in their C- terminal region. The TPα has a wide spread tissue distribution in humans and is implicated in pathophysiological conditions such as platelet aggregation, bleeding disorders, cardiovascular diseases, atherosclerosis, and asthma [2], [4], [5].

GPCRs are known to function even in the absence of an agonist molecule and this phenomenon is known as constitutive receptor activity. It can be explained using the multiple state model of receptor activation [6], [7]. Over the last decade considerable number of GPCRs were shown to have constitutive activity [8], [9]. This phenomenon of GPCRs became the most important tool in discriminating between inverse agonists and neutral antagonists [10], [11]. Inverse agonists are compounds or drugs known to reduce the constitutive GPCR activity and are often defined to have a (−1) efficacy whereas neutral antagonists do not affect the basal GPCR activity and have (0) efficacy [12]. Interestingly, a number of drugs currently in use that target GPCRs are inverse agonists rather than neutral antagonists. For example, the antagonist metoprolol for β-adrenergic receptor, losartan for Angiotensin receptor, haloperidol for Dopamine receptor and cetirizine and cimetidine for Histamine H_1_ and H_2_ receptor are now classified as inverse agonist for their respective targets [7], [12], [13], [14].

**Figure 1:**
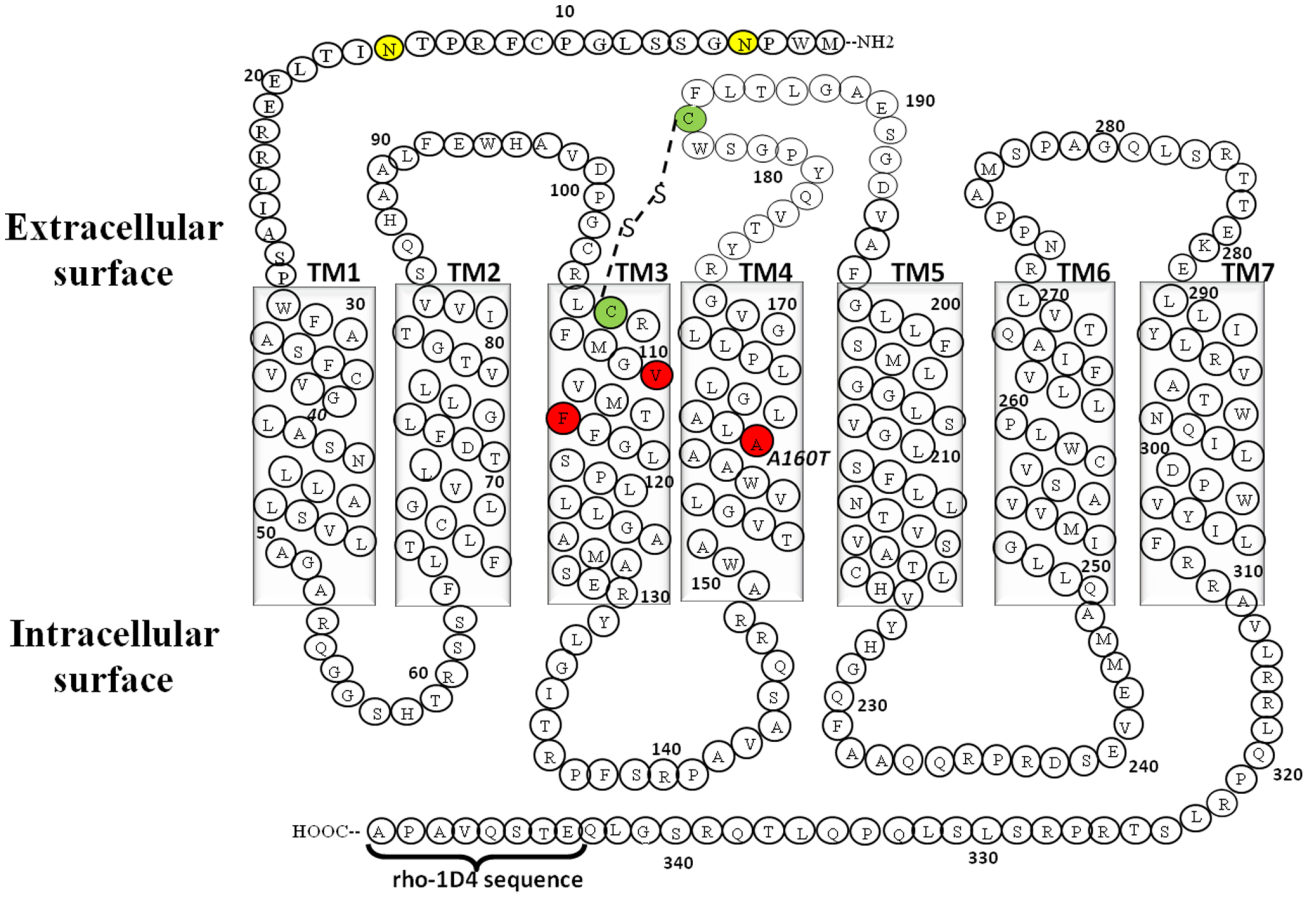
Secondary structure representation of TPα amino acid sequence. Amino acids are shown in single letter codes, and the residue numbers exclude the epitope tag (FLAG-tag) at the N-terminus. Shown are the seven transmembrane helices (TM1-7), the N-glycosylated residues Asn4 and Asn16 (yellow colored residues), the disulphide bond between Cys 105 and Cys 183 (green colored) and the rho-1D4 tag at the C -terminus. The genetic variant A160T ^4.53^ on TM4 and V110 ^3.30^, F114 ^3.34^ on TM3 (Ballesteros-Weinstein numbering in the superscript) is highlighted in red.

TP exhibits basal or constitutive activity in the absence of any ligand [15]. Previously, we have discovered constitutively active mutants (CAMs) in transmembrane (TM) 3 and 4 of TP (**Figure 1**). The mutants V110A, F114A inTM3 and the genetic variant A160T in TM4 displayed constitutive activity to varying levels [15]. Due to the excessive agonist independent activity of A160T, we speculated that this genetic variant might cause cardiovascular disease (CVD) progression [16]. For effective therapeutic intervention, an inverse agonist would be required to lower the activity of the constitutively active receptor.

TP antagonists are known to be beneficial for treating cardiovascular diseases, platelet disorders, and asthma [17], [18]. The discovery of CAMs in TP provided a unique opportunity to screen well known TP antagonists for inverse agonist activity. In this work, we chose four compounds, SQ 29,548, Ramatroban (BAY-u3405), Diclofenac and L-670596 to test for inverse agonism based on their potency and selective effects on human platelets. SQ 29,548 is a selective TP antagonist recognized for its well-established effect to antagonize platelet aggregation and contraction in respiratory smooth muscle cells [19]. Ramatroban, a TP antagonist recognized to inhibit platelet aggregation induced by collagen and U46619 [20]. Diclofenac, a non-steroidal anti-inflammatory drug (NSAID), concentration dependently and selectively inhibited TP mediated contraction in smooth muscles as well as human platelet aggregation [21]. The fourth compound we tested was L-670596, a potent TP antagonist in human platelets and shown to inhibit contraction in guinea pig tracheal rings in a concentration dependent manner [22]. We tested the pharmacological profiles of these compounds using the CAMs, in both, HEK29T cells, and in a novel megakaryocyte based system to evaluate their effects on human platelet function. Our results suggest that SQ 29,548 and Ramatroban are inverse agonists for TP, whereas, L-670596 and Diclofenac are neutral antagonists.

## Materials and Methods

The TP antagonist SQ 29,548 was purchased from Sigma (product no. NET936). Ramatroban and Diclofenac were purchased from Cayman Chemicals (Michigan, USA), L-670596 from Tocris Bioscience (Bristol, UK). The TP agonist U46619 was purchased from the Cayman Chemicals Company. Protease inhibitors and common chemicals were purchased from either Fisher or Sigma. The buffers and detergents were the same as those used previously [15]. Polyclonal antibody directed towards 1−15 amino acids of the N-terminal in TPα was purchased from Lifespan BioSciences (Washington, USA). PE-anti-CD41 antibody and FITC-anti-CD62P were purchased from Biolegend (California, USA). Nucleofection kit for Meg −01 cells was purchased from Lonza (Texas, USA).

### Molecular biology and cell culture

The TPα mutants used in this study were described previously [15]. To minimize variations in transfection efficiency, the total amount of transfected DNA was kept constant in all cases at 6 µg of DNA per 5×10^6^ cells. For transient transfections of HEK293T cells using the plasmid pMT4, lipofectamine 2000 (Invitrogen) mediated transfection was used as described by the manufacturer.

### Flow cytometry analysis of cell surface receptor expression

Cell surface expression of the WT-TP, V110A, F114A and A160T mutants transfected with different concentrations (3− 9 µg) of DNA per 5×10^6^ cells was determined using BD FACS Canto flow cytometer, and as described previously [15]. In brief, the following parameters for flow cytometry were used, a forward scatter of (132), side scatter of (313) and Alexa-488 (343).

The results shown are from a minimum of three sets of experiments. FACS data was normalized to WT-TP DNA of 3 µg which was taken as 100% (Figure S1).

### Determination of Ca ^2+^ mobilization

The DNA coding for the WT-TP, V110A, F114A and A160T were expressed in HEK 293T cells using 6 µg of DNA per 5×10^6^ cells. Changes in intracellular calcium were measured by using the fluorescent calcium sensitive dye Fluo-4NW (Invitrogen) as described previously [15], [23], [24]. Mock transfected (with vector pMT4) were used as a negative control. Determination of basal Ca^2+^ levels for agonist-independent signaling was carried out using Flexstation-3 fluorescence plate reader (Molecular Devices, CA, USA) at 525 nm following excitation at 494 nm. To determine whether the drugs decrease the basal activity of WT-TP and the mutants, cells expressing the receptors were incubated with 1 µM concentration of each of the compounds, SQ 29,548, Ramatroban, L-670596 and Diclofenac separately for 15−20 mins and the changes in intracellular calcium mobilization was determined. Ca^2+^ mobilized (ΔRFU) was corrected for receptor expression levels using FACS data. Similarly, a concentration dependent Ca^2+^ response was also measured with highest concentration of the ligands (10 µM) and lowest being buffer or water alone.

### Determination of inositol-1, 4, 5-trisphosphate (IP_3_) mobilization

IP_3_ assays were carried out in HEK293T cells using a commercially available IP_3_ assay kit (HitHunter IP_3_ fluorescence polarization [FP] assay; DiscoveRx, Fremont, CA) according to the instructions supplied by the manufacturer and as described previously [24]. A standard graph was constructed using different concentrations of IP_3_ provided by the manufacturer, and this graph was used to calculate the amount of IP_3_ released by the wild type and mutant receptor, as previously described [24]. Briefly, to determine whether the drugs decrease the basal activity of WT-TP and the mutants, cells expressing the receptors were incubated with 1 µM concentration of the compounds, SQ 29,548, Ramatroban, L-670596 and Diclofenac separately for 15−20 mins and the basal level of IP_3_ mobilization was determined and corrected for receptor expression levels using FACS data.

### Flow cytometry analysis of P-selectin (CD62P)

Human megakaryocytes (Meg-01, ATCC: CRL-2021), were nucleofected using Lonza kit C with 3 µg of WT-TP or A160T per 100,000 cells following recommended manufacturers protocol and as described previously [16]. Briefly, nucleofected cells were incubated for 24 hours at 37°C. Then platelet like particles (PLPs) was collected from the media of the nucleofected megakaryocytes. PLPs were incubated vehicle (buffer or water alone) for 15 mins at room temperature. The PLPs were incubated in PBS containing PE-anti-CD41 to label all PLPs and FITC-anti-CD62P to label activated particles and incubated for 1 hour at 4°C. The samples were washed 2 times with PBS spun down and resuspended in PBS for Flow cytometry analysis. Similarly, to assess whether the drugs decrease CD62P activation in PLPs, 1 µM concentration of each compounds, SQ 29,548, Ramatroban, L-670596 and Diclofenac on WT-TP and A160T were tested.

### Statistical analysis

Statistical analysis using one-way analysis of variance (ANOVA) with Tukey’s *post hoc* test from at least 3 independent experiments was done to determine statistical significance wherever applicable.

## Results

### Characterization of constitutive activity of WT-TP, V110A and F114A

**Figure 2:**
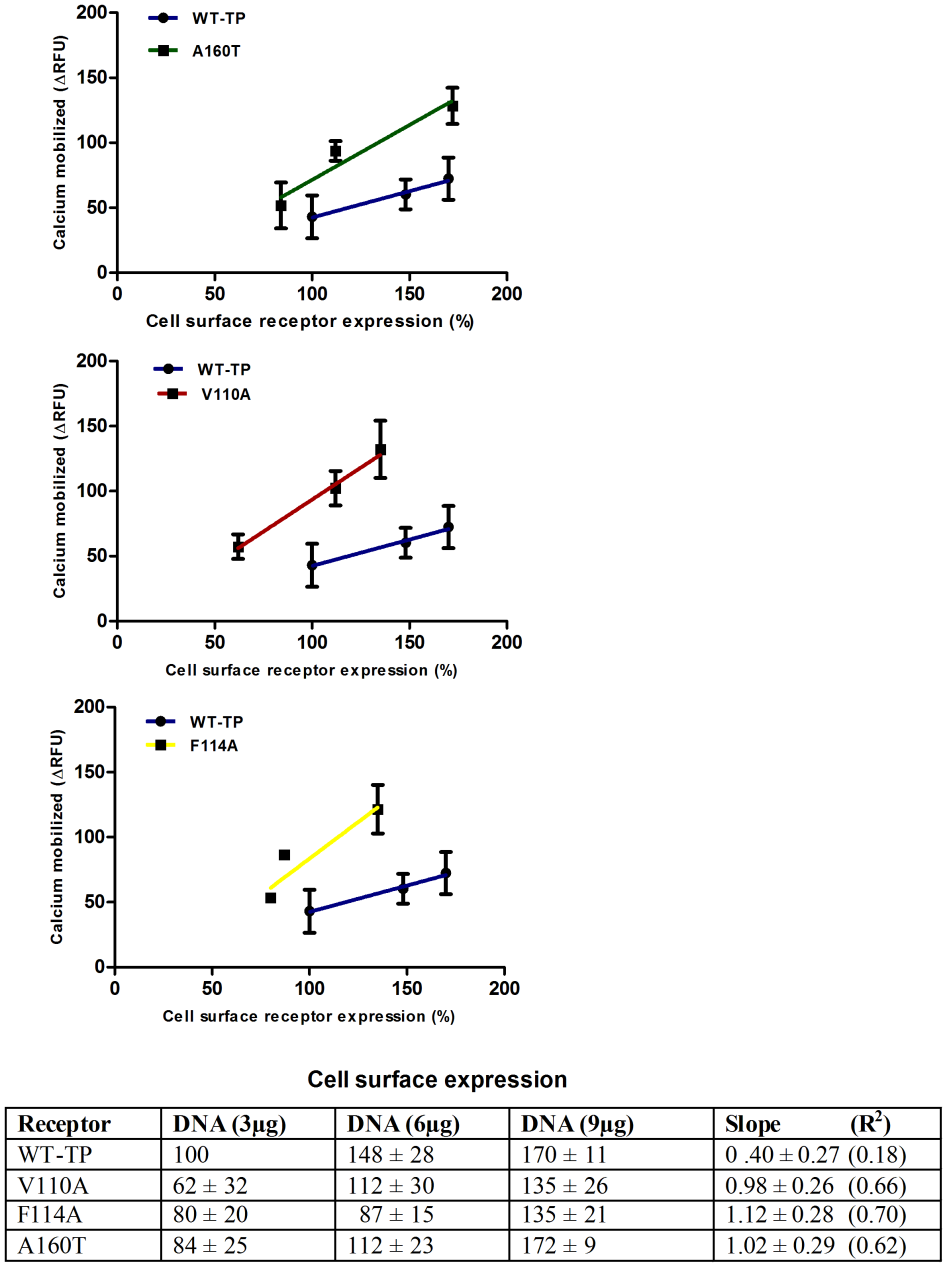
Effect of receptor density on basal Ca^2+^ mobilization. WT-TP (blue) and A160T (green), V110A (red) and F114A (yellow) constructs were expressed in HEK293T cells at different receptor densities by varying amounts of DNA used in each transfection (3 µg to 9 µg DNA per 5×10^6^ cells). Receptor expression levels were determined by FACS analysis using polyclonal antibody specific to the N-terminal sequence between amino acids 1−15 of WT-TP. FACS data was normalized to WT-TP DNA of 3 µg which was taken as 100%. The slopes of WT-TP, A160T, V110A, F114A are 0.40±0.27, 1.02±0.29, 0.98±0.26 and 1.12±0.28 respectively.

In our previous report we characterized the constitutive activity of only the A160T genetic variant, in detail [15]. We now provide detailed characterization of the constitutive activity of V110A and F114A, demonstrating the effect of receptor density on Ca^2+^ mobilization **(** **Figure 2** **)**. Analysis of the expression of the WT-TP and mutants was pursued using flow cytometry, and the specificity of the antibodies used was reported in our previous study [24]. We observed a positive linear correlation between the amounts of receptor expressed and the basal Ca^2+^ mobilization for all the mutants compared to WT-TP **(** **Figure 2** **)**. The slope of expression vs. basal activity for the V110A, F114A and A160T mutants showed high basal signaling, with constitutive activity ranging from 2 to 3 fold over WT-TP **(** **Figure 2** **)**.

### Effect of TP antagonists on constitutive intracellular Ca^2+^ signaling

**Figure 3:**
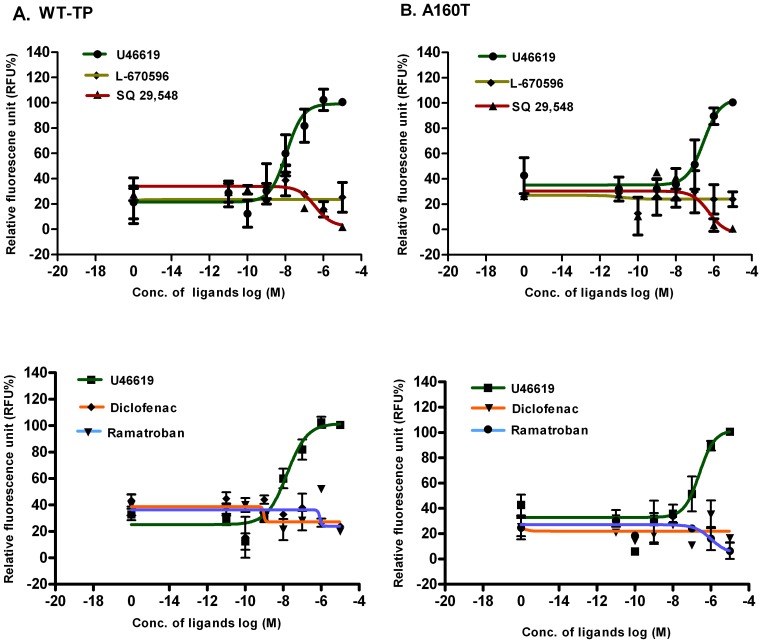
Effect of TP ligands on intracellular Ca^2+^ signaling. Concentration dependent changes in calcium mobilization of cells expressing WT-TP (A) and A160T (B) after application of different ligands. Ca**^2+^** levels were measured as described in materials and methods. Results are presented as % RFU of the maximal response obtained with after stimulation with 10 µM of TP agonist U46619. Data are represented as mean± SD and are from at least three independent experiments done in duplicate.

**Figure 4:**
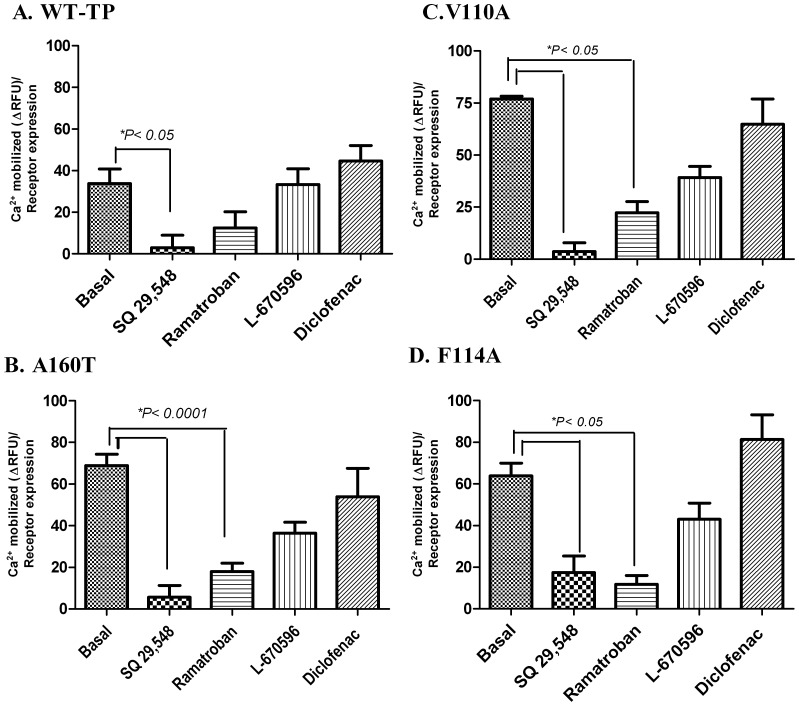
Effect of TP antagonists on constitutive intracellular Ca^2+^ signaling. Agonist-independent calcium mobilization for WT-TP, A160T, V110A and F114A after cells were pre-treated with 1 µM of SQ 29,548, Ramatroban, L670596 and Diclofenac. Ca^2+^ mobilized (Δ RFU) was corrected for receptor expression levels using FACS data. A one way ANOVA with *tukey's post hoc* test between control and mutant receptors treated with different compounds showed a significant decrease in basal activity at p<0.0001 and p<0.05. Similar results were obtained for WT-TP, basal vs SQ 29,548 at p<0.05. The results are from 3 independent experiments done in triplicate and are represented as Mean ±SD.

The concentration dependent effects of the four TP antagonists, on calcium mobilization was tested on both WT-TP and A160T, and TP agonist U46619 was used as a positive control **(** **Figure 3** **)**. SQ 29,548 was able to reduce the basal activity of WT-TP at only the two higher concentrations of 1 µM and 10 µM, whereas Ramatroban, Diclofenac, and L-670596 did not affect the basal activity of WT-TP **(** **Figure 3A** **)**. With the A160T variant, both SQ 29,548 and Ramatroban were able to reduce the basal calcium mobilization, whereas L-670596 and Diclofenac did not show any effect at higher concentrations **(** **Figure 3B** **)**. Next, we characterized the effects of the four TP antagonists, on the basal Ca^2+^ mobilization by the three CAMs. Based on our results (**Figure 3A and B**), we chose 1 µM of the compounds for our studies. The results show, 1 µM of SQ 29,548 decreased the basal Ca^2+^ mobilization of WT-TP by more than 50% whereas the other drugs did not show any statistically significant decrease **(** **Figure 4A** **).** The effect of the drugs on basal calcium mobilization of the genetic variant A160T, clearly indicate that 1 µM of SQ 29,548 and 1 µM of Ramatroban were able to decrease the basal Ca^2+^ mobilization by almost 80% and 70% respectively. However, no statistically significant change in the basal calcium mobilization was observed when treated with 1 µM of L-670596 or Diclofenac **(** **Figure 4B** **).** Similar to the results obtained with A160T, 1 µM of SQ 29,548 and 1 µM Ramatroban were able to decrease the basal activity of V110A and F114A CAMs significantly, whereas L-670596 or Diclofenac did not change the basal activity of the CAMs **(** **Figure 4C and D** **).**

### Effect of TP antagonists on intracellular IP_3_ mobilization

**Figure 5:**
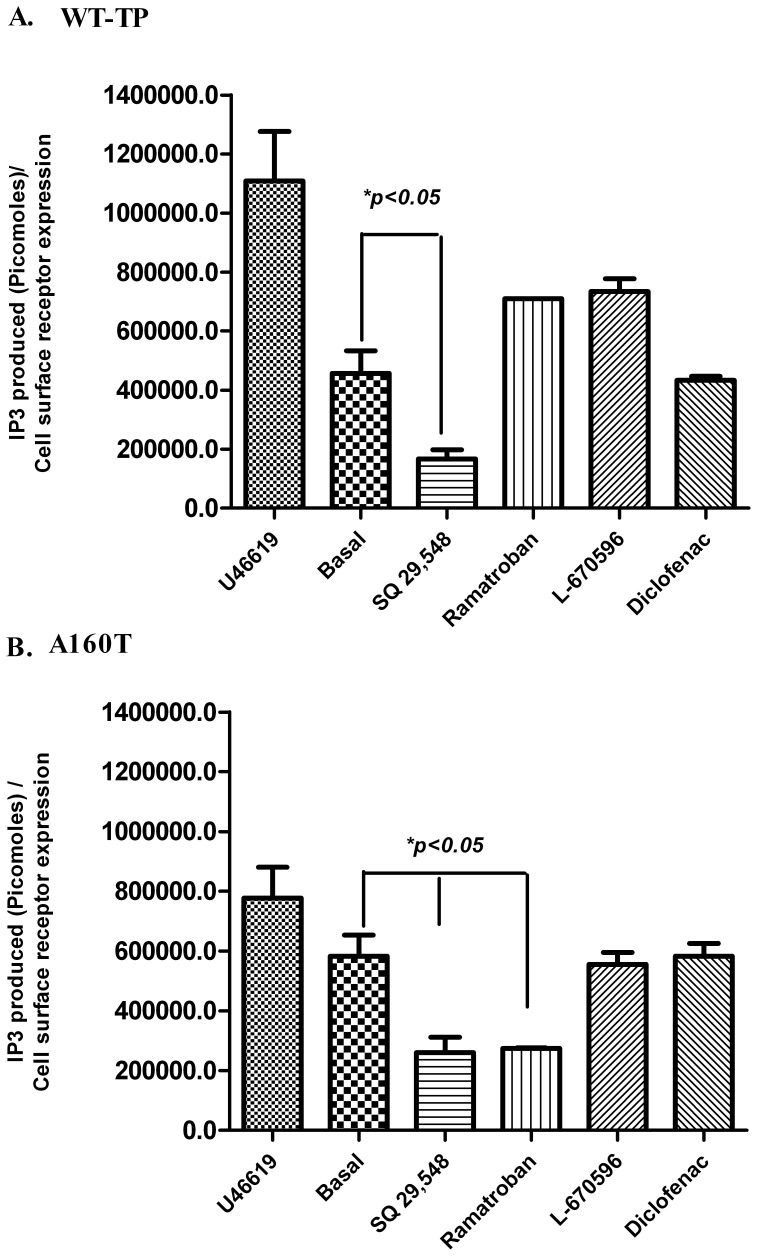
Effect of TP antagonists on intracellular IP_3_ mobilization. The bar plot diagram shows agonist U46619 (1 µM) induced and agonist-independent IP_3_ mobilization for WT-TP and A160T. The graph also shows IP_3_ release after cells were pre-treated with 1 µM of SQ 29,548, Ramatroban, L670596 or Diclofenac. Total IP_3_ (picomoles) mobilized was corrected for receptor expression levels. A one way ANOVA with *tukey's post hoc* test between basal and receptor pretreated with different compounds showed a significant decrease in basal activity at p<0.05.

The genetic variant and CAM A160T was selected for further analysis. The effects of the four TP antagonists on constitutive IP_3_ mobilization by WT-TP and A160T were studied. The TP agonist U46619 (1 µM) was used as a positive control. SQ 29,548 was able to decrease the basal activity of WT-TP by 40−50% however no statistically significant effects were observed for the other three compounds **(** **Figure 5A** **)**. Interestingly, 1 µM of SQ 29,548 or Ramatroban, were able to decrease the basal activity of A160T by 50% **(** **Figure 5B** **).** However, no significant decrease in the basal IP_3_ mobilization was observed when WT-TP or A160T were treated with 1 µM of L-670596 or Diclofenac.

### Effect of TP antagonists “In Platelet” functional analysis

**Figure 6:**
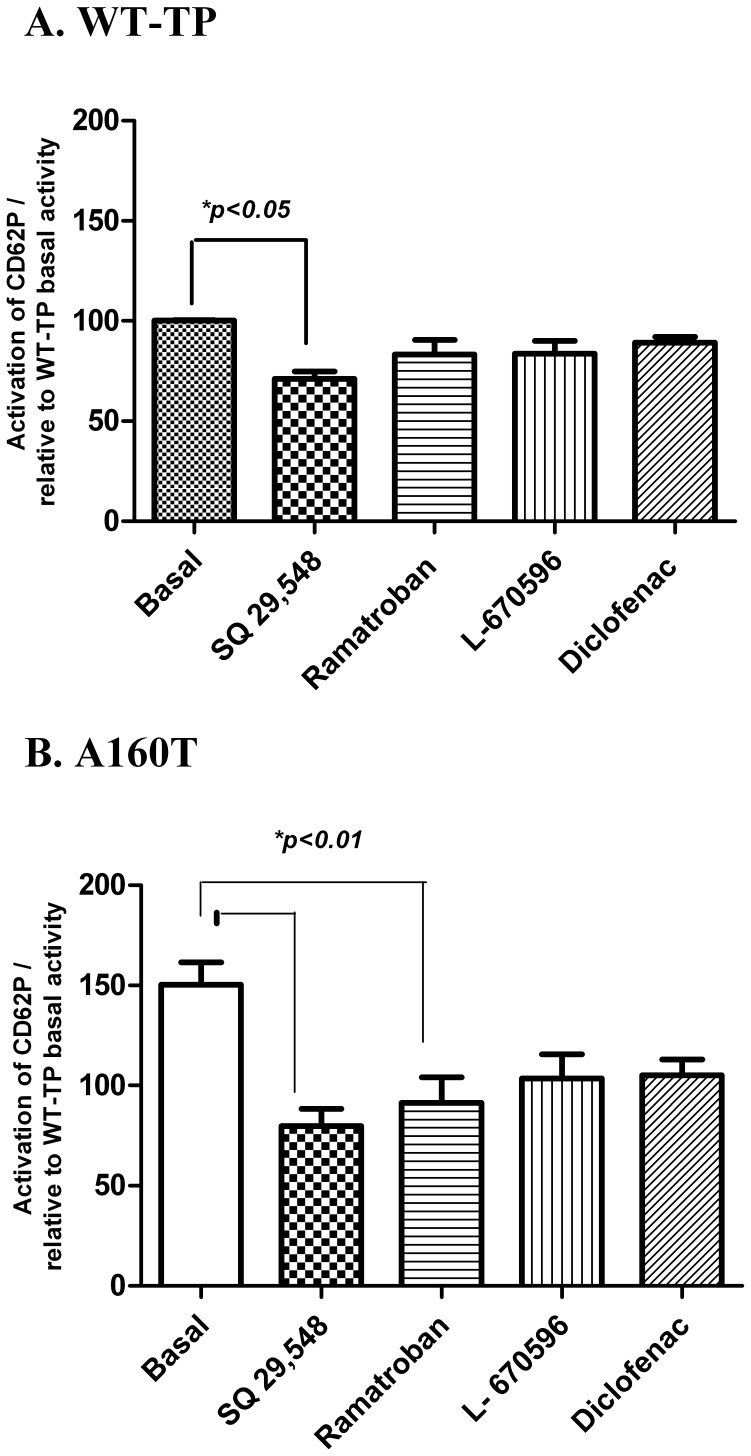
Effect of TP antagonists “In Platelet” Functional Analysis. The bar plot represents FACS analysis of P selectin (CD62P) on the surface PLPs liberated from cultured Meg-01 cells. Activity or response under basal conditions were measured. A160T showed a considerable higher basal activity compared to that of WT-TP, which was decreased by addition of each of 1 µM SQ 29,548 or Ramatroban. A one way ANOVA with *tukey's post hoc test* between WT-TP and SQ 29,548 as well as A160T pretreated with different compounds SQ 29548, and Ramatroban showed a significant decrease in basal activity at p<0.05 and p<0.01 respectively. The results are from a minimum of 3 independent experiments and are represented as Mean ± SD.

Studies involving identification of inverse agonist(s) for GPCRs have routinely used heterologous expression systems. To have a more physiologically acceptable scenario, we used a human platelet like system that can be genetically modified. Recently, we have shown that Meg-01 can be transfected and can produce platelet like particles [16]. To test the inverse agonist effect of the drugs on the constitutive activity of A160T in Meg-01 cells, the genetic variant A160T was transfected into the Meg-01 cells and PLPs collected. WT-TP was used as the negative control. PLPs were measured by flow cytometry for CD62P (P-selectin) after basal activation (i.e.) in presence of buffer alone (control) and after the use of the drugs SQ 29,548, Ramatroban, L-670596 and Diclofenac. The A160T variant, as expected showed higher baseline activity than WT-TP **(** **Figure 6** **)**. The results from the heterologous system were once again validated, when it was found that both 1 µM of SQ 29,548 as well as Ramatroban decreased the basal activity of A160T significantly, whereas 1 µM of L-670596 and 1 µM of Diclofenac had no significant effect on the basal activity of A160T **(** **Figure 6** **)**. This finding is clinically important as it demonstrates the inverse agonist properties in our PLP model, suggesting there may be a similar protective effect against cardiovascular disease *in vivo*.

## Discussion

Over expression of GPCRs in some cases has been shown to enhance the basal receptor activity, which in turn facilitates understanding of ligand interactions with its receptor and the signal transduction pathway [7], [25]. The concept of constitutive receptor activity is now well established and is often caused due to the presence of single nucleotide polymorphisms (SNPs) or mutations in TMs or intracellular loop domains. SNPs are defined to occur in at least 1% of the population and are often linked with receptor disorder or human diseases [26]. Some of these SNPs can lead to human diseases, while others can induce multiple signaling states in the receptor leading to distinct signaling pathways. In rhodopsin, the G114V^3.29^ and Q184P^4.73^ (Ballesteros and Weinstein numbering used [27]) variants have been shown to cause protein misfolding resulting in retinitis pigmentosa in patients [28]. Similarly, the V103I^2.63^ mutation in the melanocortin receptor has been associated with decreased incidence of obesity in normal individuals [29]. Interestingly, *in vitro* studies of the R347C variant in the C-terminus of α1A-adrenoreceptor showed that the mutant did not affect receptor signaling and trafficking mechanism, which is consistent with *in vivo* data that showed the variant in humans as predicted was not related to any human disease [30]. Other examples include melanocortin-4 receptor (MC4R)-CAMs, thyroid stimulating hormone (TSH)-CAMs, and luteinizing stimulating hormone (LSH)-CAMs which are linked to different human diseases [26], [31]. Our recent studies from sequencing 897 cardiovascular patients did not reveal the presence of the A160T^4.53^ variant [16]. Though the clinical significance of the A160T in TP is not yet elucidated we speculate that this SNP, because of its constitutive activity might cause CVD progression or involved in other pathophysiological process.

In this report, we confirmed a positive correlation between receptor density and basal calcium released, establishing that V110A^3.30^ and F114A^3.34^ are CAMs. Following transfection of F114A, V110A, A160T and WT-TP, the basal calcium mobilization was decreased when treated with 1 µM of SQ 29,548 or 1 µM of Ramatroban demonstrating their role as inverse agonists, whereas no effect was observed for Diclofenac or L-670596 treatment. The inverse agonist activity of SQ 29,548 and Ramatroban was also confirmed by IP_3_ mobilization assays.

In the next part, we focused on the SNP variant A160T in TP. Previous studies have shown that rodent thromboxane system differs significantly from the human system [16], [32]. To investigate the role of the four compounds in an accepted physiological scenario such as human platelet function, we tested their effects on Meg-01 based system for platelet activation. Meg-01 cells spontaneously release PLPs into culture medium which express markers such as CD41 and CD62P on their surface [33]. Since P selectin (CD62P) are only expressed on activated platelets, measuring CD62P expression on platelet surface using flow cytometric assay has been widely employed to characterize platelet activation in various experimental and clinical conditions [34]. Our data using the Meg-01 revealed that there is a spontaneous over expression of P selectin on the PLPs containing A160T compared to WT-TP without any agonist treatment leading to constitutive activity. Strikingly, the constitutive activity was decreased for A160T when treated with 1 µM concentration of SQ 29,548 or Ramatroban validating their role as inverse agonists **(** **Figure 6** **)**. However, no significant effect was observed for Diclofenac and L-670596 suggesting their role as neutral antagonist.

A TP antagonist or inverse agonist would preferably be more acceptable to low dose aspirin in the light of the recent events surrounding COX-2 inhibitors [35]. Previous studies have highlighted the clinical side effects of COX-2 inhibitors, as the TXA2/PGI2 balance is critical in maintaining the cardiovascular homeostasis [4], [35]. The pharmacological characterization of TP CAMs, allowed us to revisit some of the potent TP antagonists and classify them under neutral and inverse agonist categories.

In conclusion, we report that SQ 29,548 and Ramatroban are inverse agonists for the A160T genetic variant of TP towards IP_3_ signaling and platelet activation. Given the crucial role played by TP in maintaining vascular homeostasis, SNPs such as A160T in TP that are also CAMs, can have significant clinical manifestations. In the light of the foreseen therapeutic relevance, identification of inverse agonists for TP could be beneficial in clinical applications.

## Supporting Information

Figure S1 **Flow cytometry analysis of cell surface receptor expression.** Mean fluorescence intensity (MFI) and percentage positive cells (raw values) for WT and the mutant receptors before normalisation are shown. The bar graph shows the MFI (raw values) calculated from three independent experiments after deducting the negative control (cells mock transfected with vector alone). The table shows the average of the percentage positive cells expressing the receptor of interest after deducting the negative control (cells mock transfected with vector alone) from three independent experiments. Data are represented as Mean ± SD. The data is normalised to the WT-TP (3 µg of DNA) taken as 100%.
(DOC)
