# Supplementary material for: Inverse Agonism of SQ 29,548 and Ramatroban on Thromboxane A2 Receptor
Source: PLoS One. 2014 Jan 23;9(1):e85937. doi: 10.1371/journal.pone.0085937 (PMC3900440; doi:10.1371/journal.pone.0085937)
Supplement: Figure S1 — Flow cytometry analysis of cell surface receptor expression. Mean fluorescence intensity (MFI) and percentage positive cells (raw values) for WT and the mutant receptors before normalisation are shown. The bar graph shows the MFI (raw values) calculated from three independent experiments after deducting the negative control (cells mock transfected with vector alone). The table shows the average of the percentage positive cells expressing the receptor of interest after deducting the negative control (cells mock transfected with vector alone) from three independent experiments. Data are represented as Mean ± SD. The data is normalised to the WT-TP (3 µg of DNA) taken as 100%. (DOC) [file pone.0085937.s001.doc]

| **Receptor** | **3µg of DNA** | **6µg of DNA** | **9µg of DNA** |
| --- | --- | --- | --- |
| WT-TP | 4.70 ± 0.4 | 5.00 ± 1.4 | 8.00 ± 2.1 |
| A160T | 2.40 ± 0.7 | 3.66 ± 0.5 | 6.42 ± 0.7 |
| V110A | 2.23 ± 0.6 | 4.28 ± 1.8 | 5.03 ± 1.9 |
| F114A | 4.20 ± 1.1 | 5.50 ± 0.8 | 6.34 ± 0.5 |

**
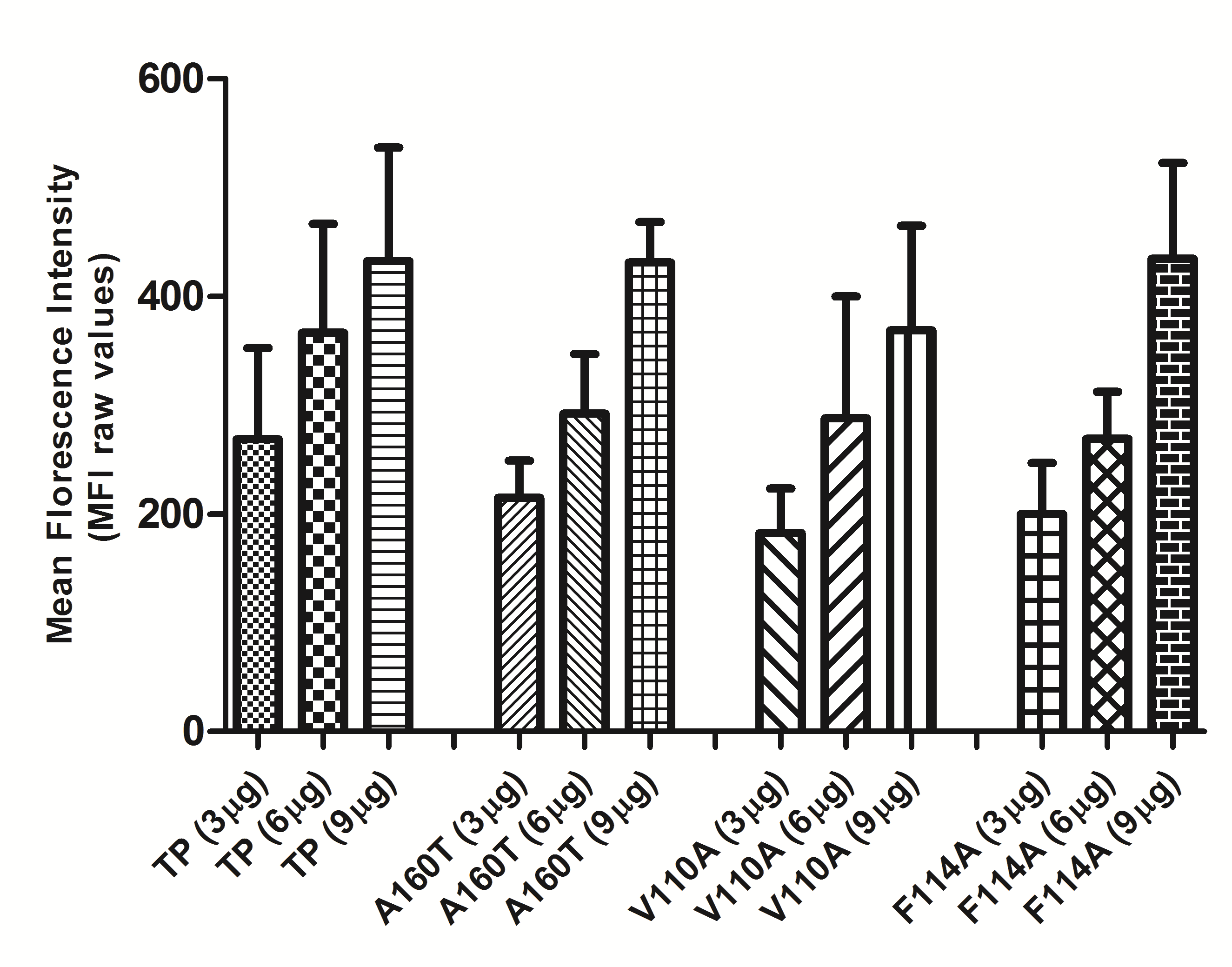
**

**Figure S1. Flow cytometry analysis of cell surface receptor expression.** Mean fluorescence intensity (MFI) and percentage positive cells (raw values) for WT and the mutant receptors before normalisation are shown. The bar graph shows the MFI (raw values) calculated from three independent experiments after deducting the negative control (cells mock transfected with vector alone). The table shows the average of the percentage positive cells expressing the receptor of interest after deducting the negative control (cells mock transfected with vector alone) from three independent experiments. Data are represented as Mean ± SD. The data is normalised to the WT-TP (3µg of DNA) taken as 100%.
